# Caution for Multidrug Therapy: Significant Baroreflex Afferent Neuroexcitation Coordinated by Multi-Channels/Pumps Under the Threshold Concentration of Yoda1 and Dobutamine Combination

**DOI:** 10.3390/biom14101311

**Published:** 2024-10-16

**Authors:** Yin-zhi Xu, Zhao-yuan Xu, Hui-xiao Fu, Mao Yue, Jia-qun Li, Chang-peng Cui, Di Wu, Bai-yan Li

**Affiliations:** 1 State Key Laboratory of Frigid Zone Cardiovascular Diseases (SKLFZCD), Department of Pharmacology (State Key Laboratory-Province Key Laboratories of Biomedicine-Pharmaceutics of China, Key Laboratory of Cardiovascular Research, Ministry of Education), College of Pharmacy, Harbin Medical University, Harbin 150081, China; 2 Department of Pharmacy, The Second Affiliated Hospital of Dalian Medical University, Dalian 116023, China; 3 Research Unit of Noninfectious Chronic Diseases in Frigid Zone (2019RU070), Chinese Academy of Medical Sciences, Harbin 150081, China

**Keywords:** aortic depressor nerve, compound action potential, baroreceptor neurons, action potential, ion channels/ion pumps

## Abstract

Multi-drug therapies are common in cardiovascular disease intervention; however, io channel/pump coordination has not been tested electrophysiologically. Apparently, inward currents were not elicited by Yoda1/10 nM or Dobutamine/100 nM alone in Ah-type baroreceptor neurons, but were by their combination. To verify this, electroneurography and the whole-cell patch-clamp technique were performed. The results showed that Ah- and C-volley were dramatically increased by the combination at 0.5 V and 5 V, in contrast to A-volley, as consistent with repetitive discharge elicited by step and ramp with markedly reduced current injection/stimulus intensity. Notably, a frequency-dependent action potential (AP) duration was increased with Iberiotoxin-sensitive K^+^ component. Furthermore, an increased peak in AP measured in phase plots suggested enhanced Na^+^ influx, cytoplasmic Ca^2+^ accumulation through reverse mode of Na^+^/Ca^2+^ exchanger, and, consequently, functional KCa1.1 up-regulation. Strikingly, the Yoda1- or Dbtm-mediated small/transient Na^+^/K^+^-pump currents were robustly increased by their combination, implying a quick ion equilibration that may also be synchronized by hyperpolarization-induced voltage-sag, enabling faster repetitive firing. These novel findings demonstrate multi-channel/pump collaboration together to integrate neurotransmission at the cellular level for baroreflex, providing an afferent explanation in sexual dimorphic blood pressure regulation, and raising the caution regarding the individual drug concentration in multi-drug therapies to optimize efficacy and minimize toxicity.

## 1. Introduction

The diversity of the functional expression of multi-channels/pumps contributes to the unique patterns in the baroreflex afferent neurocontrol of circulation and multi-drug therapies, which are common in cardiovascular disease intervention. It is well known that neuronal signaling is encoded into the frequency of action potential (AP) within spike train [1] that subsequently leads to the presynaptic release of corresponding neurotransmitters known as central integration. Later observations have demonstrated that the integration can also be presented at the peripheral level such as in the cell bodies of neurons through use-/frequency-dependent AP duration (APD) [2,3]. Diversity in the functional expression of ion channels, such as Piezo1, voltage-gated Na^+^ (Nav1.7, 1.8, and 1.9), Ca^2+^ (N-type), Ca^2+^-activated K^+^ (KCa1.1), and HCN (HCN1), contributes to the unique patterns of activity generated in visceral afferent neurons, including baroreceptor neurons (BRNs), in response to their natural stimuli [4]. The Na^+^/Ca^2+^ exchanger (NCX) and Na^+^/K^+^-pump are also crucial for transmembrane ion homeostasis and neuronal excitation, not only in the central nervous system (CNS) [5], but also in the peripheral nervous system (PNS) [6]. These ATP-driven pumps induce the movement of those ions to re-establish the appropriate ionic balance of ions inside and outside of excitable membrane. Consequently, it is easy to understand that the alternation of these channel/pump activities would definitely impact the neuronal excitability through the modulation of membrane depolarization/repolarization. Compiling evidence suggests that NCX and Na^+^/K^+^-pump are expressed in vagal neurons and play an essential role in the regulation of visceral reflexes [6,7] including baroreflex, which promotes cardiac vagal activity, leading to the anti-hypertensive effect [8]. The Na^+^/K^+^-pump is also stimulated by β1-adrenergic signaling [9,10], Piezo1 activation [11,12], and is co-played with HCN1 [13]. Apparently, both ion channels and pumps are perfectly tuned in vivo during neurotransmission, which is triggered upon stimulations such as changes in visceral reflexes (visceral pain)/baroreflex (hypertension) or medications. However, visualizing this collaboration is challenging. Therefore, in the current investigation, neuronal excitability was evaluated by compound AP using electroneurography (ENG) from the aortic depressor nerve (ADN) in intact female rats, and AP discharge properties related to Na^+^, KCa1.1, and HCN, as well as NCX and Na^+^/K^+^-pump currents, were measured using the whole-cell patch technique in the presence of Yoda1, Dobutamine (Dbtm), alone, or in combination, in identified Ah-type BRNs isolated from adult female rats. These novel findings, together with our previous evidences [2,14], have revealed at least in part that Piezo1, KCa1.1, HCN, NCX, and Na-K-pump coordinate together to integrate neuroexcitation at the cellular and presynaptic levels of Ah-type baroreflex afferents and explain the sexual dimorphic neurocontrol of circulation. This also raises caution regarding individual drug concentration used for multi-drug therapies to offer better pharmacological efficacy with possible lower toxicity.

## 2. Materials and Methods

### 2.1. Animals

Adult female Sprague-Dawley (SD) rats, weighing approximately 200 ± 20 g, were provided by the Animal Center of the Second Affiliated Hospital of Harbin Medical University (SCXK (Hei) 2019-001) for ENG, AP, and ion channel current recordings from neurons isolated from the nodose ganglion (NG). The rats were housed under standard conditions, as previously described [14], for at least for one week prior to experimentation. All protocols involving the animals used for in vivo or in vitro studies were pre-approved by the Institutional Animal Care and Use Committee of Harbin Medical University. These protocols adhere to the recommendations of the American Veterinary Medical Association’s Panel on Euthanasia and the National Institutes of Health publication “Guide for the Care and Use of Laboratory Animals (http://www.nap.edu/readingroom/books/labrats/ accessed on 30 December 2022)”.

### 2.2. Chemicals

Chemicals including Yoda1 (#HY-18723) and NS-11021 (#HY-131003) were obtained from MedChemExpress (Monmouth Junction, NJ, USA). Iberiotoxin (#I5904), Kb-r7943 (#K4144), Capsaicin (Cap, #211275), and Dobutamine (Dbtm, #D0676) were all acquired from Sigma-Aldrich (St. Louis, MO, USA). These drugs were dissolved in appropriate solvents before use. All chemicals used in recording solutions, as well as enzymes required for neuronal isolation, were purchased from standard commercial sources.

### 2.3. Compound AP Recordings

The ENG recording protocol for intact rats was as previously described [15]. Initially, rats were fully sedated to ensure complete relaxation using the combination (i.p.) of urethane (800 mg/kg) and α-chloralose (80 mg/kg) for thein situ study of baroreflex. Subsequently, the left aortic depressor nerve (ADN) was delicately dissected, paying particular attention to avoid damaging two adjacent tiny arteries. Both the stimulus and recording electrodes were then positioned on the ADN, spaced to maximize the distance necessary for acquiring compound APs with minimal delay following the stimulus artifact. ENG data were captured following a series of voltage stimulations at a controlled body temperature of 34–35 °C. This was conducted in the presence of Yoda1, Dbtm, and their combination. The elicited responses were quantitatively assessed using the root mean square (RMS) method.

### 2.4. Neuron Isolation and Afferent Fiber Type Identification

The procedures for neuron isolation from adult rats have been described in detail previously [16,17]. Briefly, under relaxation using a mixture of ketamine/xylazine (0.1 mL/100 g, i.p.), the neurons acutely isolated from adult female rats were cultured for at least 4 h prior to recordings, allowing for recovery of ion channels from enzymatic treatment. All recordings were completed within 6 to 10 h following isolation.

### 2.5. Afferent Fiber Type Identification

The identification of afferent fiber types in BRNs isolated from the NG of adult female rats was achieved through the use of the electrophysiological characteristics of AP. These waveform characteristics include the AP duration at 50% repolarization (APD_50_), maximum upstroke velocity (UV_MAX_), and maximum downstroke velocity (DV_MAX_), along with the presence of a hump over the time course of repolarization. This identification was complemented by pharmacological sensitivity to Capsaicin [16] and confirmed by positive fluorescence following Dil labeling on ADN fibers in 4-week-old female rats [18].

### 2.6. Whole-Cell Patch Recordings

Whole-cell patch recordings were conducted using both voltage- and current-clamp modes to measure single AP and repetitive discharges evoked by brief pulse, as well as depolarization current steps or ramps and to assess voltage-gated K^+^ and Na^+^/K^+^-pump currents. These procedures adhered to previously described protocols [16,17] and were carried out at room temperature (22–23 °C) on a PC platform utilizing P-Clamp software (v10.3, Molecular Devices; Sunnyvale, CA, USA). In this study, younger female SD rats weighing approximately 75 g underwent ADN labeling with Dil (Eugene, OR, USA), following our routine protocols [19]. The preparation of recording electrodes, the composition of recording solutions, and the detailed protocols for patch-clamp recordings have been elaborated in previous publications [16,17].

### 2.7. Statistical Analysis

Statistical analysis and graph creation were conducted using Excel v2007 and Origin v7.0 software (Microsoft, Northampton, MA, USA). All electrophysiological data generated by patch-clamp recordings were analyzed with Clampfit software (v10.3, Molecular Devices; Sunnyvale, CA, USA). Statistical analyses were only performed on studies that had at least four complete observations (*n*), as indicated in the figure legends. The normal distribution of data was automatically tested during the creation of graph with Origin. Both paired or unpaired the Student’s *t*-test were employed to assess significant differences before and after treatment or between groups, as appropriate. When necessary, one-way ANOVA with post-hoc Tukey test was utilized. The averaged data are presented as mean ± SD. A *p* value of less than 0.05 was considered statistically significant.

## 3. Results

### 3.1. Effects of Yoda1, Dbtm, and Their Combination on Inward Currents Recorded in Identified Baroreceptor Neurons Isolated from Adult Female Rats

**Figure 1:**
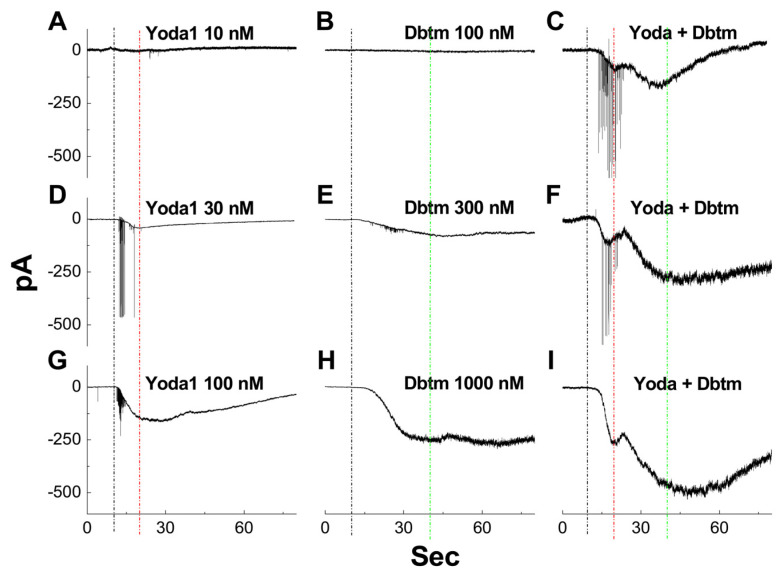
Inward currents recorded in the identified Ah-type baroreceptor neurons using gap-free protocol under voltage-clamp mode before and after Yoda1 (10, 30, 100 nM), Dobutamine (Dbtm, 100, 300, 1000 nM), and the combination. The recording was held at −60 mV for 120 s. (**A**,**D**,**G**): Concentration-dependent effects of Yoda1 on inward currents. (**B**,**E**,**H**): Concentration-dependent effects of Dbtm on inward currents. (**C**,**F**,**I**): Concentration-dependent effect of Yoda1/Dbtm combination on inward currents. The black dot dash line: time to application; red dot dash line: the peak time of Yoda1; green dot dash line: peak time of the combination.

To investigate the potential effects of Yoda1 and Dbtm on baroreceptor neuroexcitation [14,20] and to determine the appropriate concentration, the inward currents were evoked using the gap-free protocol (Figure S1) under the voltage-clamp mode with −60 mV holding before and after the bath administration of Yoda1 (10, 30, and 100 nM) and Dbtm (100, 300, and 1000 nM), as well as their combination. The results revealed that concentration-dependent inward currents in the presence of Yoda1 (Figure 1A,D,G) or Dbtm (Figure 1B,E,H) were observed, with peak currents occurring approximately 5 s after Yoda1 administration and 40 s after Dbtm, accompanied by a high frequency of spikes during the initial phase of inward currents, particularly for Yoda1. Interestingly, even though detectable inward currents were not observed under the threshold concentration of either Yoda1/10 nM (Figure 1A) or Dbtm/100 nM (Figure 1B), significant inward currents were evoked by their combination (Figure 1C, applied simultaneously through bath perfusion) with clear double current peaks observed at close to 5 s and 40 s that were similar to those in the case of Yoda1 or Dbtm applied separately. This alternation could further be confirmed by the combined use of higher concentrations of Yoda1/Dbtm (Figure 1F,I) with significantly amplified inward currents peaking at a similar time frame in a concentration-dependent manner. This dataset strongly suggests that inward currents may be synergistically potentiated by the combination of Yoda1 and Dbtm. To further elucidate these effects, we will investigate neuroexcitability involving multiple ion channels and pumps/exchangers using Yoda1 at 10 nM, Dbtm at 100 nM, or their combination in subsequent in vivo or in vitro investigations.

### 3.2. Effect of Yoda1, Dbtm, or Their Combination on Compound AP from the Intact Aortic Depressor Nerve of Adult Female Rats Using Electroneurography

**Figure 2:**
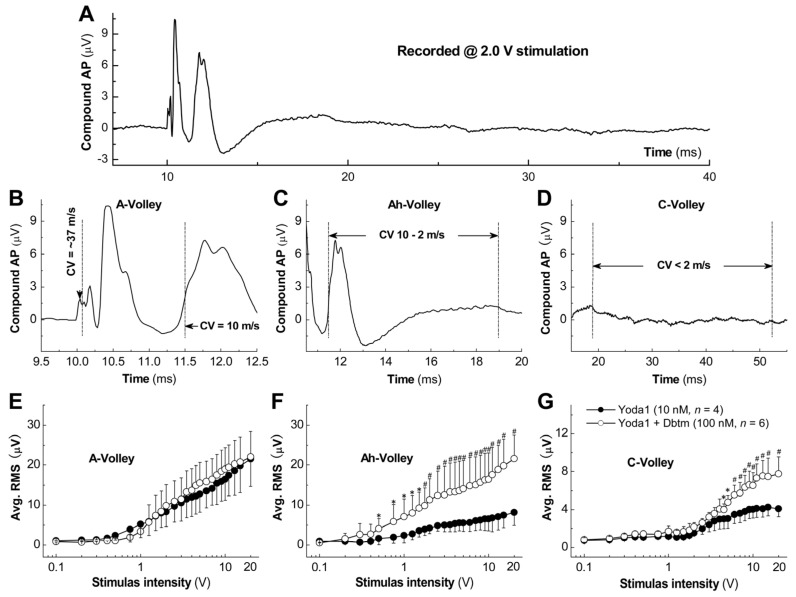
Changes in compound action potential (AP) recorded from aortic depressor nerve (ADN) in intact adult female SD rats in the presence of Yoda1 or Yoda1 alone with Dobutamine (Dbtm). Compound AP was elicited by bi-polar electrode using a series of voltage ranged from 0.1 to 20 V and averaged root mean square (RMS, μV) was calculated. A-volley, Ah-volley, and C-volley represented the composition of all A-, Ah-, and C-type baroreceptor afferents, respectively, and were identified according to the afferent fiber conduction velocity (time from stimulation to the waveform/length of ADN, m/s). (**A**): Representative recording with 2.0 V stimulation. (**B**–**D**): Representative A-volley (>10 m/s), Ah-volley (2–10 m/s), and C-volley (<2 m/s) between each paired vertical dash dot line; downward arrowhead shown in (**B**) means the stimulus artifact. (**E**–**G**): Averaged (Avg.) RMS for (**B**–**D**). Unpaired *t*-test was used between groups, data were presented as mean ± SD, and *n* = 4 and 6 for Yoda1 (10 nM) and Yoda1 + Dbtm (100 nM); * *p* < 0.05 and ^#^ *p* < 0.01 vs. Yoda1 at the same time point.

Our previous observations have shown that Yoda1 mediates, dose dependently, BP reduction in both adult male and age-matched female rats [14], wherein a female-specific subpopulation of myelinated Ah-type BRNs may play a role in facilitating baroreflex neurotransmission at both the cellular and presynaptic levels. Electroneurography (ENG) was employed by applying a series of voltage stimulations to the ADN from intact female rats and recording compound AP (Figure 2), which was then quantified by root mean square (RMS) analysis. Based upon our pilot study (Figure 1), the concentration of Yoda1 10 nM and Dbtm 100 nM were chosen for ENG testing. Despite negative effects on inward currents observed at the cellular level, both agents induced detectable compound APs of similar amplitude, with their averaged RMS more than doubling compared to controls (Figure S2). This suggests that ENG may be more sensitive for monitoring baroreflex afferent function in response to these agents as opposed to BP measurement.

To distinguish A- and Ah-volley (compound AP) without the interference of C-volley and potential noise, 2.0 V-evoked ENG was demonstrated (Figure 2A). Upon the simultaneous administration of Yoda1 and Dbtm, A-volley appeared immediately following the stimulus artifact (Figure 2B, arrowhead), with fiber conduction velocities ranging from 10 to 37 m/s, succeeded by Ah-volley at 2–10 m/s (Figure 2C). Notably, A-volley exhibited greater uniformity, with the majority of conduction centered at 27–30 m/s compared to Ah-volley, which had a broader conduction range, likely due to a wider distribution of myelin and the properties of myelinated axons [16,21]. Remarkably, average RMS values indicated a significant increase in Ah-valley due to the Yoda1 and Dbtm combination across a range of 0.5–20 V stimulus intensities (Figure 2F), unlike A-volley (Figure 2E). Moreover, an increased C-volley under similar conditions could only be evoked at stimulus intensities exceeding 5 V (Figure 2D,G and Figure S3), aligning with its unmyelinated characteristic and higher AP discharge threshold [16]. These observations strongly suggest that Ah-type baroreflex afferents exhibit a broad range of myelin distribution and diverse conduction speeds, as well as heightened chemosensitivity and neuroexcitability, making their role in baroreflex afferent neurotransmission crucial, and potentially more amenable to modulation and targeting by pharmacological treatments.

### 3.3. Effect of Yoda1, Dbtm, or Their Combination on Repetitive Discharge of AP Elicited by Step Current Depolarization in Identified Ah-Type BRNs

**Figure 3:**
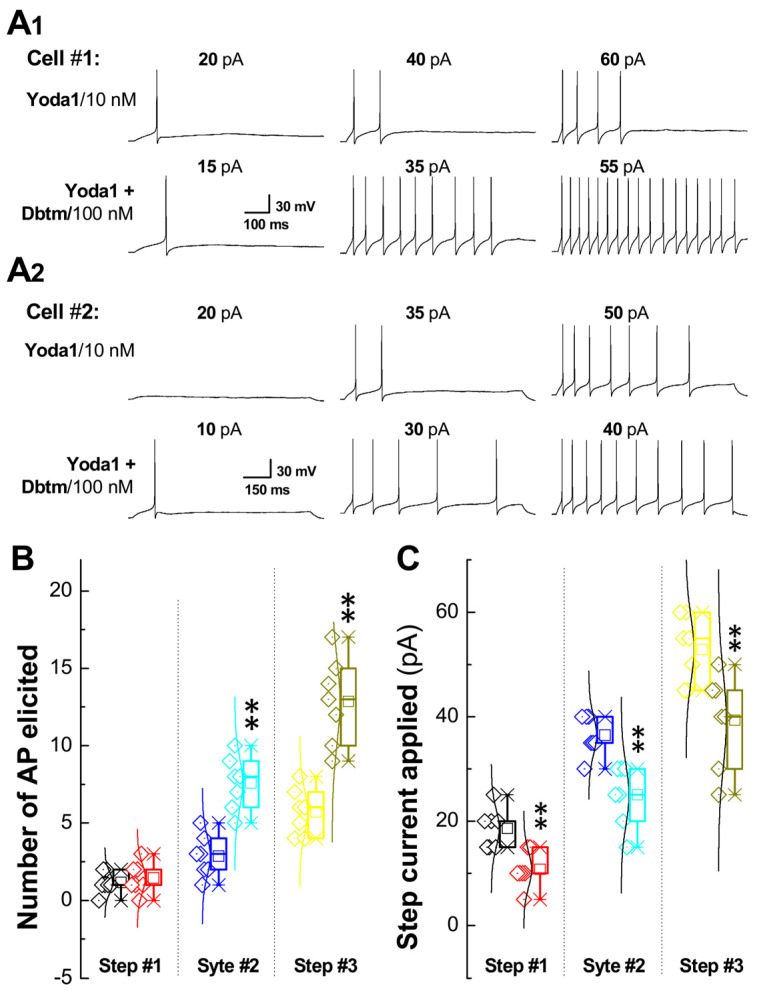
Changes in discharge capability of action potential (AP) recorded from identified Ah-type baroreceptor neurons (BRNs) isolated from adult female SD rats in the presence of Yoda1 or Yoda1 + Dobutamine (Dbtm). AP was elicited by stepped current injection under voltage-clamp mode of whole-cell patch configuration. (**A1**,**A2**): Representative recordings were obtained from two different Ah-type BRNs (Cell #1 @ the top two rows and Cell #2 @ the bottom two rows with three steps for each treatment) in the presence of Yoda1 10 nM (as control) or Yoda1 + Dbtm 100 nM. (**B**): Summarized data of the number of AP elicited within each step before (black/step #1, blue/step #2, and yellow/step #3) and after treatment (red/step #1, light blue/step #2, and green/step #3). (**C**): Summarized data of the step current applied for step. Repetitive discharges were collected before and after Dbtm and paired *t*-test was applied. Averaged data were expressed as mean ± SD, *n* = 7. ** *p* < 0.01 vs. Yoda1. Scaled bars also applied for other step recordings of the same cell.

In these experiments, Ah-type BRNs were identified by their fluorescence [18] and discharge characters [16,21], such as low AP firing threshold, shorter APD_50_, and a faster rate of the derivatives of depolarization and repolarization conjugated with repolarization hump (Figure S4). Initially, the effects of either Yoda1/10 or Dbtm/100 on AP repetitive discharge were tested using a step current depolarization protocol (Figure S5). No statistical differences were found compared with untreated data or between the groups tested (Figure S6), which is consistent with the observed negative effects on inward currents (Figure 1A,B). However, the simultaneous application (via dual-channel perfusion) of Yoda1 and Dbtm significantly increased the numbers of APs in the spike train as stimulus intensity was raised (Figure 3A1,A2, two sets of representative recordings, Figure 3B), with a robustly reduced step current required (Figure 3C), suggesting a synergistic potentiation of the Yoda1/Dbtm combination on the capability for repetitive discharge.

**Figure 4:**
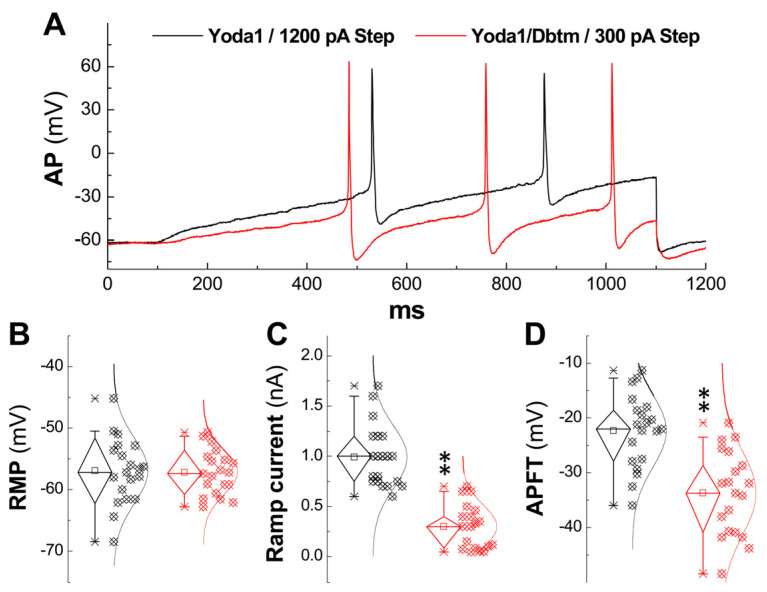
Changes in the ramp current applied for evoking similar AP discharge in identified Ah-type BRNs in the presence of Yoda1 or Yoda1 + Dbtm. To determine the excitability under similar AP discharge by ramp protocol, the ramp current was quantified in identified Ah-type BRNs. (**A**): Representative recordings in the presence of Yoda1 (10 nM, black) and Yoda1 + Dbtm (100 nM, red). (**B**): Summarized data for the resting membrane potential (RMP). (**C**): Summarized ramp current applied. (**D**) Summarized APFT. Unpaired *t*-test was selected between groups and averaged data were expressed as mean ± SD, *n* = 20 for Yoda1, and *n* = 24 for Yoda1 + Dbtm. ** *p* < 0.01 vs. Yoda1.

To further verify the synergistic or potentiated effect of this combination on repetitive discharge, a ramp protocol was also utilized (Figure S7). The proper ramp current injection was chosen to maintain discharge profiles similar to those of the tested Ah-type BRNs. The averaged data indicated that the combination elicited a nearly identical frequency of repetitive discharge of APs (Figure 4A), led to a decreased APFT (*p* < 0.01, Figure 4C), and required a significantly reduced ramp current (*p* < 0.01, Figure 4D) compared with Yoda1 alone as the control (*n* = 20). This occurred without detectable effects on the resting membrane potential (RMP, Figure 4B) between the two datasets.

Upon closer examination of the AP trajectory, the afterhyperpolarization peak was significantly heightened following the combination treatment, suggesting a possible enhancement of hyperpolarization-activated, cyclic-nucleotide-gated (HCN), channel-mediated sag potential [22,23]. Consequently, a specific step protocol was applied (Figure S8 left) and the results demonstrated that, compared with Yoda1 as control, both the peaked/sustained sag potential (trace 1) and repetitive AP discharge (Trace 4) were markedly increased (Figure S8 right) by the combination, along with the increased peak of afterhyperpolarization (AHP), peak of AP, and APFT (Figure S8 bottom table).

### 3.4. Effect of Yoda1/Dbtm Combination on the AP Waveform/Trajectory in the Spike of Ah-Type BRNs

**Figure 5:**
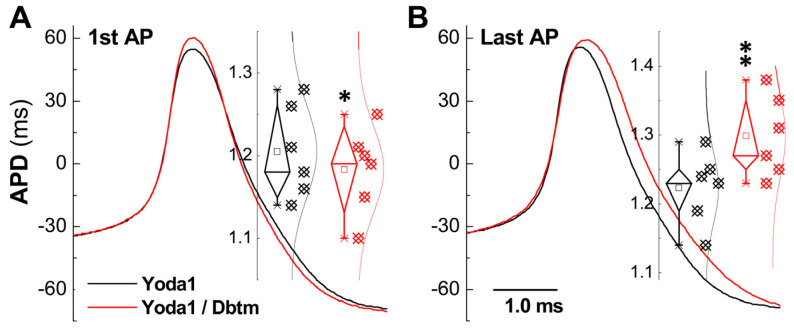
Alternative changes in action potential duration (APD_50_) of identified Ah-type BRNs in the presence of Yoda1 or Yoda1 + Dbtm. The first (1st) and the last APs in the spike trains of repetitive discharges in the presence of Yoda1 and Yoda1 + Dbtm shown were superimposed; resting membrane potential (RMP), APD_50_, and the peak of AP were measured accordingly: (**A**): the 1st APs, (**B**): the last APs. Averaged data were expressed as mean ± SD, *n* = 6 complete recordings. * *p* < 0.05 or ** *p* < 0.01 vs. Yoda1. The horizontal bar in the (**B**) was also applied for (**A**).

Given the enhanced capability of repetitive discharge, the KCa1.1 channels are likely to be implicated, with frequency-dependent AP broadening expected in the presence of Yoda1 or the Yoda1/Dbtm combination. An analysis of APD_50_ of both the first and last AP showed an average increase of 1.5% (*p* = 0.05, from 1.205 ± 0.056 to 1.223 ± 0.052 ms) with Yoda1 alone and a more substantial 9.72% (*p* < 0.01, from 1.18 ± 0.05 to 1.29 ± 0.05 ms) with the combination. Surprisingly, by the superimposition of the first (Figure 5A and *inset*) or last APs (Figure 5B and *inset*) in the spike train (Figure 3A1,A2) revealed that the average of APD_50_ of the first AP when treated with the combination was actually narrowed by about 3.38% (*p* = 0.031 vs. Yoda1). In contrast, the APD_50_ of the last AP was significantly prolonged by approximately 6.13% (*p* = 0.0008 vs. Yoda1), indicating the functional up-regulation of KCa1.1, leading to a shortened APD_50_ of the first AP and gradual inactivation during the spike train, resulting in frequency-dependent AP broadening.

**Figure 6:**
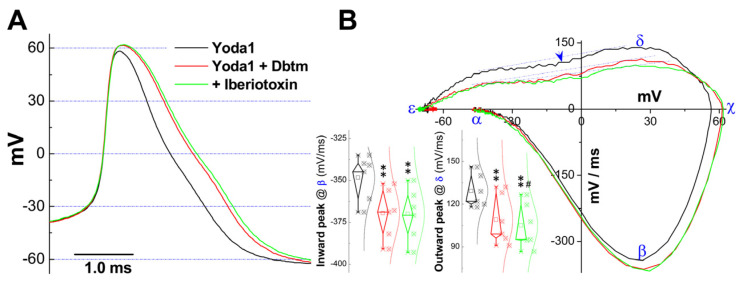
Iberiotoxin/KCa1.1 inactivation abolishes AP widening induced by Yoda1 + Dbtm in identified Ah-type BRNs. Repetitive discharge of AP was elicited by step depolarization in the presence of Yoda1 and Yoda1 + Dbtm without/with Iberiotoxin, and complete recordings in one cell were included for further analysis. (**A**): The last APs in the spike trains were superimposed. (**B**): Phase plots: the total membrane current plotted as a function of membrane voltage from each AP shown in (**A**), and α, β, χ, δ, and ε were represented for the AP firing threshold, the maximal up-stroke velocity of total inward current/depolarization phase (negative portion), the peak of AP, the maximal down-stroke velocity of total outward current/repolarization phase (positive portion), and the peak of hyperpolarization, respectively; blue arrowhead means the location of the repolarization humps: *Inset/left and inset/right*: changes in total inward and outward. Averaged data were presented as mean ± SD, *n* = 6 complete recordings from at least four preparations, ** *p* < 0.01 vs. Yoda1, ^#^ *p* < 0.01 vs. Yoda1 + Dbtm.

As this point, the question arises as to what would occur if KCa1.1 channels were blocked in relation to frequency-dependent AP broadening. To investigate this, the repetitive discharge of APs was recorded in identified Ah-type BRN neurons with or without 100 nM Iberiotoxin (IbTx) applied via micro perfusion directly onto the tested cell in addition to the Yoda1/Dbtm combination. Notably, the increased repetitive discharge rate remained similar, while the frequency-dependent AP broadening was absent. However, the APD_50_ observed in the last AP was slightly widened (Figure 6A), but showed no difference with the presence of IbTx compared to the combination alone, presumably due to the minor change in APD and insufficient sensitivity of the analysis.

Rather than focusing solely onAPD, phase plotting was employed as a more sensitive method to evaluate all waveform characteristics (Figure 6B). As anticipated, several detailed alternations were detected; the results demonstrated significant alterations in the total currents behind depolarization (Figure 6B, β, *insets on the left*) caused by the combination, which remained unchanged with the addition of IbTx. Moreover, the peak of AP, as revealed by the plots (Figure 6B, γ), provided further support for the synergistic/potentiated effect of Yoda1/Dbtm combination, which was also not affected by IbTx. More definitively, the total outward current behind repolarization was further reduced (Figure 6B, δ, *insets on the right*) by IbTx (*p* = 0.043 vs. combination), even though significant differences were not reported by measuring APD (Figure 6A). This dataset strongly suggests that KCa1.1 and voltage-gate Na^+^ currents are major contributors to the synergistic/potentiated effect of the Yoda1/Dbtm combination. There is increasing evidence that more Na^+^ influx during depolarization can activate the reverse mode of the NCX, resulting in intracellular Ca^2+^accumulation [24,25,26], which subsequently leads to further functional up-regulation of KCa1.1 through a coupling mechanism.

### 3.5. Na^+^ Influx Mediated Intracellular Ca^2+^Mobilization, Likely through the Reverse Mode of Na^+^/Ca^2+^Exchanger NCX, Which Contributes to KCa1.1 Functional Expression in Identified Ah-Type BRNs

Clearly, the peak amplitude of the AP was substantially increased throughout the spike train, as shown after the superimposition of the last traces from two separate recordings (Figure S9), corresponding to step (Figure 3A) and ramp protocol (Figure 4A). The averaged peak amplitude of the AP in both the first and last AP, and post-combination treatment, was significantly increased by approximately 7.65% and 8.04%, respectively, when compared to treatment with Yoda1 alone (*p* < 0.01). These findings suggest that the current behind depolarization has likely increased, possibly due to the combination [27] that functionally up-regulates the current density of Na^+^. It is widely accepted that an increased Na^+^ influx through voltage-gated channels during depolarization leads to the recruitment of additional K^+^ currents, including KCa1.1, during repolarization.

There is mounting evidence to suggest that the reverse mode of NCX can be activated by this Na^+^ influx, leading to an accumulation of cytoplasmic Ca^2+^ that subsequently reinforces the functional expression of KCa1.1. To elucidate whether NCX plays a role in APD modulation, repetitive discharge triggered by the Yoda1/Dbtm combination was analyzed before (Figure S10A) and after the application of KB-r9437 100 nM (Figure S10C), a specific antagonist for NCX. The results clearly indicated that not only was the number of APs within the spike train reduced, but frequency-dependent AP broadening was also blocked by KB-r7943 (Figure S10B,D), as evidenced by the superimposition of three individual APs (first, center, and last in the spike train). This phenomenon may be explained by several factors: (1) When the frequency of repetitive discharge is slow in the case of KB-r7943, allowing membrane potential to return to resting state, use-dependent Na^+^ channel inactivation may not occur during the spike train evidenced by a similar AP firing threshold, the rate of depolarization, and the peak of AP. (2) Blocking the reverse mode of NCX with KB-r7943 inhibits not just the NCX, but also the Na^+^ currents, leading to a synergistic effect on recruitment of K^+^ currents, including KCa1.1. (3) For the Na^+^/K^+^ pump, along with hyperpolarization-induced voltage sag, there may be additional factors promoting the shaping of repolarization for rapid transmembrane ion equilibrium.

### 3.6. IbTx-Sensitive Components as a Key Player to Regulate Neuronal Excitability/Integration at the Cellular Level by Shaping Repolarization/Frequency-Dependent APD Prolongation in Identified Ah-Type BRNs

**Figure 7:**
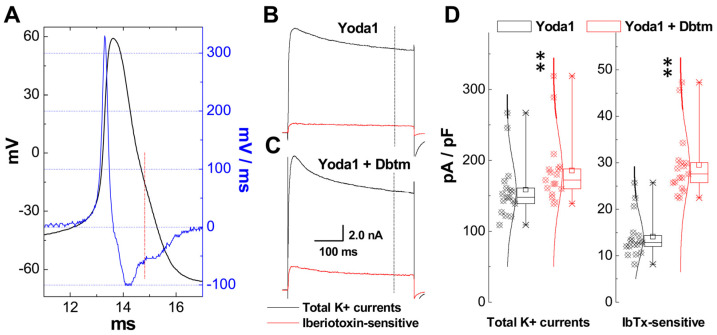
Effects of Iberiotoxin (IbTx) on total K^+^ currents in the presence of Yoda1 + Dbtm recorded from identified Ah-BRNs. After AP was recorded for afferent fiber type identification, the extracellular solution was changed with bath perfusion to the one for potassium current recording; the cell was clamped at a holding potential of −80 mV and stepped from −70 mV up to +40 mV with 5 mV increment with an interval of 1 s between sweeps. Potassium currents were recorded in the presence of Yoda1/10 nM, Dbtm/100 nM, and Yoda1 + Dbtm, respectively. IbTx 100 nM was micropurfused to the tested cell to avoid contaminating other cells in the chamber after successfully recording the total K^+^ currents, and IbTx-sensitive components were obtained by subtraction. Total current was divided by its whole-cell capacitance and current density was presented as pA/pF. (**A**): Representative recording of Ah-type BRN identified by waveform characters, the vertical dash dot line means the presence of repolarization hump. (**B**,**C**): Representative tracings of total and IbTx-sensitive K^+^ currents. Scale bars also applied for (**B**). (**D**): Summarized data for comparisons of total and IbTx-sensitive components. Averaged results were presented as mean ± SD, *n* = 19 recordings from at least nine preparations; ** *p* < 0.01 vs. Yoda1.

Our previous findings have conclusively shown that afferent neural signals can be integrated at the cellular level through a mechanism of frequency-dependent AP prolongation, which leads to Ca^2+^ influx at the presynaptic membrane and neurotransmitter release in unmyelinated C-type neurons, as opposed to myelinated A-types [3,28]. Further research has also emphasized a female-specific distribution of myelinated Ah-type visceral/baroreceptor neurons with the functional expression of KCa1.1 [2,14,15,16,19,21,29]. Consequently, we firmly believe that the neuronal excitation and frequency-dependent events mediated by the combination are due to the functional up-regulation of KCa1.1, which is facilitated by elevated cytoplasmic Ca^2+^ through both the reverse mode of NCX and the combination itself [30,31]. As anticipated, the current density of both the total outward K^+^ currents (Figure 7B) and IbTx-sensitive components (Figure 7C), along with the averaged data (Figure 7D), showed significant enhancement (*p*< 0.01 vs. totals) in identified Ah-type BRNs (Figure 7A) due to the combination treatment. To corroborate this change, the current density of total K^+^ currents in the presence of either Yoda1 or Dbtm alone was also calculated, revealing no statistical differences (Figure S11).

To further verify the involvement of the KCa1.1/IbTx-sensitive component, a separate set of repetitive discharges was analyzed, and the last APs collected in the presence of Yoda1, the combination, or plus NS11021 100 nM were superimposed (Figure S12). The data revealed that, similar to the effects of KB-r7943, the APD_50_ broadening (Figure S12A) and increased outward peak (negative portion, Figure S12B) induced by the combination were not only reversed by NS11021, but also further increased (*p* < 0.01 vs. combination) in outward peak (Figure S12C) and showed a reduction (*p* < 0.01 vs. combination) in APD_50_ (Figure S12D). These datasets indicate that KCa1.1 is instrumental in modulating AP trajectory and neuroexcitation under physiological conditions and can be up-regulated by the Yoda1/Dbtm combination.

### 3.7. Activation of Na^+^/K^+^-Pump in the Presence of Yoda1/Dbtm Combination: A Way to Manipulate Neuronal Excitability by Accelerating Transmembrane Ion Equilibration in Identified Ah-Type BRNs

**Figure 8:**
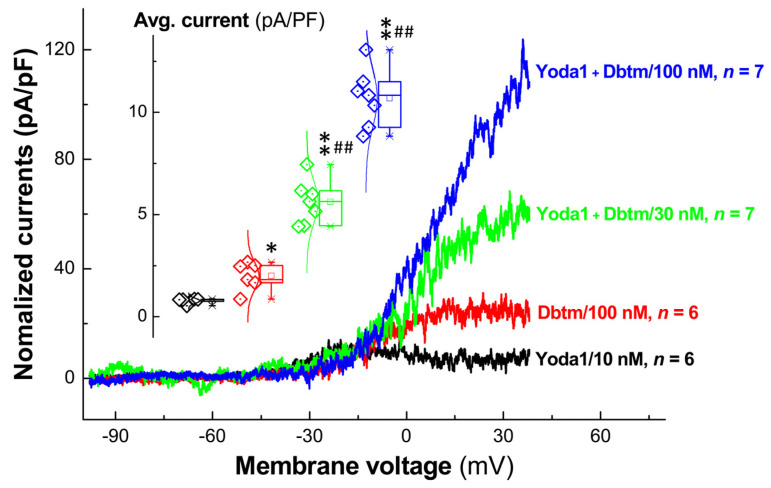
Changes in Na^+^-K^+^-ATPase currents recorded in identified Ah-type BRNs in the presence of Yoda1 or Yoda1 + Dbtm. Na^+^-K^+^-ATPase currents were recorded under physiological condition with intracellular concentration of 8.9 mM and extracellular concentration of 145 mM. The order of the recording was Yoda1 (10 nM, black, *n* = 6), Dbtm (100 nM, red, *n* = 6), Yoda1 + 30 nM Dbtm (green, *n* = 7), and Yoda1 + 100 nM Dbtm (blue, *n* = 7). Inset showing the summarized analysis. Unpaired *t*-test was applied between groups, and one-way ANOVA was also tested among groups with post-hoc Tukey test. Averaged data were expressed as mean ± SD; * *p* < 0.05 and ** *p* < 0.01 vs. Yoda1; ^##^ *p* < 0.01 vs. Dbtm.

In the field of electrophysiology, it is well understood that the Na^+^/K^+^-pump is pivotal for modulating the excitability of excitable cells by maintaining ion gradients, neuroexcitability, the propagation of APs, and electro-mechanical coupling in the visceral/baroreflex afferent system. Consequently, we examined the currents mediated by the Na^+^/K^+^-pump in identified Ah-type BRNs induced by Yoda1, Dbtm, and their combination using a specific ramp protocol (Figure S13). The results indicated that both Yoda1 and Dbtm alone generated modest and transient Na^+^/K^+^-pump currents. Remarkably, these currents increased more than tenfold when treated with the Yoda1/Dbtm combination and even quintupled when the Dbtm concentration was reduced to 30 nM within the combination (Figure 8). This suggests that the amplified Na^+^/K^+^-pump currents facilitate rapid ion equilibration back to the resting state, which could explain the observed increase in the frequency of repetitive firing with the combination treatment. Obviously, this cellular phenomenon cannot be simply attributed to a synergistic or potentiated action, and further exploration to elucidate the underlying mechanisms is warranted.

## 4. Discussion

Neuroexcitability is a key focus in neuroscience, and is significant for understanding both normal physiology and the pathophysiology underlying various diseases. It is acknowledged that neural signaling, such as baroreflex afferent responses to blood pressure fluctuations, is encapsulated in the action potential (AP) frequency of baroreceptor neurons (BRNs). This signaling is integrated at multiple levels, modulating firing frequency and, consequently, frequency-dependent action potential duration (APD). Such changes can facilitate greater Ca^2+^ influx at the presynaptic membrane, thereby inducing more intense neurotransmitter release. Thus, the AP is fundamental to grasping neuroexcitability, but the intricate interplay among various ion channels and pumps that shape the AP trajectory is complex and not fully understood, with scant literature available. Commonly, investigations into neuroexcitation focus on the effects of single ion channel modulation by pharmacological means under both physiological and disease conditions. This singular approach may hinder researchers and clinicians from discovering precise treatments with optimal dosages that provide maximum efficacy with minimal side effects. In this project, the neuroexcitation of baroreflex afferents was evaluated using ENG in intact rats and patch-clamp techniques in isolated Ah-type baroreceptor neurons. The threshold concentration (showing no detectable effect) of Yoda1 10 nM (Piezo1 agonist), Dbtm 100 nM (β-receptor agonist), or their combination was applied to quantify repetitive discharge and frequency-dependent APD modulation and the ions channels/pumps insights related to AP depolarization/repolarization, including Na^+^, Ca^2+^-activated K^+^/KCa1.1, HCN-mediated sag potential, and Na^+^ influx-induced reverse mode of NCX activation, as well as Na^+^/K^+^-pump current, providing a clearer understanding of how these channels and pumps collaborate together to regulate neuroexcitability.

Baroreflex afferent inputs, arising from the ADN, directly respond to BP. Thus, the excitation of these afferents can be analyzed in vivo under appropriate stimulation before and after treatment. The compound APs obtained using ENG from adult female rats represent the myelinated A- and Ah-, as well as unmyelinated C-component (term as volley). Within this framework, the averaged RMS for the Ah-volley was significantly increased by the combination starting from 0.5 V (Figure 1C,F) and was less significant for C-volley (Figure 1D,G) with a higher voltage stimulation at 5V. However, the difference in the A-volley (Figure 1B,E) between Yoda1 alone and the Yoda1/Dbtm combination was not observed. This in vivo result adds compelling evidence that both the significance/distribution of Ah-type afferents [2,14,16,18,21] and their inputs play a critical role in baroreflex afferent function, showing substantial sensitization to sexual-dimorphism-related conditions such as hypertension [32,33], asthma [34,35,36], silent angina [37,38], and others.

To further understanding the mechanisms of neuroexcitation, identified Ah-type BRNs isolated from adult female rats, coupled with whole-cell patch-clamp studies, serve as the optimal model at the cellular level to elucidate the presumed coordination of multi-channels/pumps supporting membrane current derivatives. While altered discharge profiles were not elicited by Yoda1 or Dbtm alone, higher frequency discharges triggered by lower step currents were indeed observed with their combination (Figure 2) in identified Ah-type BRNs, correlating well with the enhanced Ah-volley from ENG under the same conditions. A close examination of the AP trajectory confirmed frequency-dependent ADP prolongation after superimposing APs before and after the combination treatment. This finding was also supported by the ramp protocol, where the current required was significantly reduced to maintain a similar discharge profile (Figure 3). In all experimental conditions, a noticeable increase in the AP peak with rapid depolarization throughout the entire spike train (Figure 4) aligned with the evidence provided by AP phase plots, demonstrating a larger negative peak of derivative (Figure 5, β) and positive peak of voltage (Figure 5, γ). This indicates that voltage-gated Na^+^ [39], Na^+^-induced Ca^2+^ influx via the reverse mode of the Na^+^/Ca^2+^ exchanger [24,25,26], and subsequent Ca^2+^-activated-K^+^/KCa1.1 currents [3,29] were significantly modified. Nonetheless, the complexity of ion channels/ion pumps coordination cannot be explained merely as a potentiated or synergistic action between Yoda1/Piezo1 and Dbtm/β-receptor activation. Therefore, blockers for Piezo1 or Dbtm were not ambitiously tested; given that the concentration of Yoda1 or Dbtm was below the threshold, a blocking effect may not be observable.

Our previously published data have demonstrated that Ah-type BRNs functionally express Nav1.7, Nav1.8, and Nav1.9 [27,40], contributing to their higher excitability. Thus, under the current experimental conditions, the use of the antagonist tetrodotoxin to determine which Na^+^ channel subtypes are involved in the regulation of the peak of AP is not straightforward, since Nav1.8 and Nav1.9 are not sensitive to the toxin, and excitability would definitely be changed due to Nav1.7 inhibition. Additionally, altering the extracellular concentration of Na^+^/Ca^2+^ is likely to change the surface charge of the membrane and consequently the excitability [41,42]; therefore, this method was not chosen to confirm the peak of AP. Despite these limitations, the increased peak inAP by the combination could be substantiated by the rate of depolarization, as the Na^+^ channel is the only one activated in this process.

The extant literature well documents that Na^+^ influx can mobilize intracellular Ca^2+^accumulation, likely via the reverse mode of the Na^+^/Ca^2+^ exchanger [24,25,26], which presumably activatesKCa1.1, primarily through the coupling mechanism between Ca^2+^ and KCa1.1 [43,44]. Further effects on KCa1.1 could be exerted by Yoda1 [45] and/or Dbtm [26,27]. This hypothesis is supported by the observation that frequency-dependent AP broadening induced by the combination is abolished by IbTx, and the maximal peak of the total outward current, as measured by phase plots, is also further reduced (Figure 5, δ) with no frequency reduction in repetitive discharge [46] or the peak of the AP. Conversely, an increase in the total K^+^ current density and IbTx-sensitive component after KCa1.1 activation with NS11021 supports the functional up-regulation of KCa1.1 in Ah-type BRNs.

The aforementioned up-regulation of KCa1.1 is ascribed to cytoplasmic Ca^2+^ accumulation, at least partly via the reverse mode of Na^+^/Ca^2+^ exchanger. Therefore, repetitive discharge was analyzed with and without KB-r7943. The results were in line with our hypothesis, showing that discharge frequency decreased by more than 50%, and frequency-dependent APD broadening was completely eliminated. This change is mainly attributed to the disruption of Ca^2+^ accumulation via the reverse mode of the exchanger, rather than to a low firing frequency, which would result in APs within the spike train depolarizing at a similar firing threshold and recruiting a comparable quantity of KCa1.1, thereby becoming inactivated during repetitive discharge.

The importance of the Na^+^/K^+^-pump in facilitating rapid transmembrane ion equilibrium during AP processes is clear. Whether the combination of Yoda1 and Dbtm enhances pump function is an intriguing question. Our exciting evidence indicated that Yoda1 or Dbtm alone prompts only minor and brief pump currents, whereas their combination markedly increases pump current in a Dbtm concentration-dependent manner. This significant upsurge in pump activity could expedite the return of ions across the cell membrane, effectively priming the neuron for subsequent excitation more rapidly.

## 5. Conclusions

**Figure 9:**
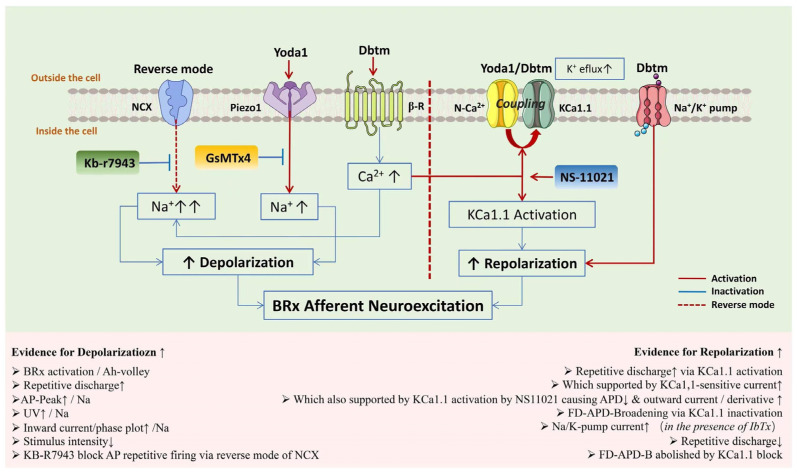
Schematic outline of up-regulated baroreflex afferent neuroexcitation through coordination of multi-ion channel and pump activation over the course of action potential by the combination use of Yoda1 and Dobutamine under threshold concentration.

In this pioneering investigation, we have explored the coordination of multi-ion channels and ion pumps during the action potential process, utilizing both electroneurography in vivo in the aortic depressor nerve and whole-cell patch-clamp techniques in vitro in identified Ah-type baroreceptor neurons of adult female rats. Our findings indicate that Yoda1 or Dobutamine alone at the tested concentrations did not have detectable effects on the neuroexcitation of baroreflex afferents, which is, however, significantly enhanced by their combination. This effect is presumably mediated through the functional up-regulation of voltage-gated Na^+^ channels, leading to intracellular Ca^2+^ accumulation, likely via the reverse operation of Na^+^/Ca^2+^ exchangers. Subsequently, there is an elevation of the current density of KCa1.1, possibly due to the coupling between voltage-gated Ca^2+^ channels and KCa1.1 on the membrane. This up-regulated KCa1.1 is suggested to be a pivotal element in mediating not only high-frequency discharge but also frequency-dependent action potential broadening through its frequency-dependent inactivation. Through this mechanism, baroreflex afferent signals could be efficiently integrated at the cellular level at the cell bodies of Ah-type BRNs, as well as at the presynaptic membrane, to enhance neurotransmitter release via prolonged action potential duration; in addition, elevated Na^+^/K^+^-pump activity and HCN-mediated faster hyperpolarization by the combination are plausible players to promote neuroexcitation via quick ion equilibration (Figure 9). It is worth noting that this increase in neuroexcitation cannot be simply attributed to potentiation or synergistic action between Piezo1 and β-receptor activation, indicating that further investigation into the complex coordination among these elements is warranted.

## Data Availability

The data that support the findings of this study are available from the corresponding author upon reasonable request.

## Supplementary Materials

The following supporting information can be downloaded at: https://www.mdpi.com/article/10.3390/biom14101311/s1, Figure S1: Gap-free protocol for inward current recording under the voltage-clamp mode with −60 mV holding. Figure S2: Electroneurography (ENG) of aortic depressor nerve (ADN) at 1.0 V stimulation (control) and in the presence of Yoda1 10 nM (Yoda1/10) or Dobutamine 100 nM (Dbtm/100). Figure S3: Compound action potential recorded by using electroneurography (ENG) of aortic depressor nerve (ADN) at 6.0 V stimulation (control). Figure S4: Action potential (AP, black) and membrane current plotted as the function of membrane voltage/derivatives (dV/dt, blue) recorded in identified myelinated Ah-type baroreceptor neurons (BRNs) isolated from adult female rats. Figure S5: The illustration of the voltage-clamp protocol for step current depolarization to elicit the repetitive discharge of action potential. Figure S6: The representative recording of repetitive discharge of action potential in the presence of Yoda1 or Dobutamine. Figure S7: The representative voltage-clamp protocols for ramp recordings used for Yoda1 10 nM and Dobutamine 100 nM alone (left) or Yoda1 10 nM + Dobutamine 100 nM (right). Figure S8: Effects of Yoda1 along (black) and combination of Yoda1/Dbtm (red) on reparative discharge and the sag potential/hyperpolarization recorded in the Ah-type baroreceptor neurons isolated from adult female rats. Figure S9: The superimposition of the last recording traces elicited by step protocol showing in the Figure 3 in the presence of Yoda1 as control and the Yoda1/Dbtm combination. Figure S10: Repetitive discharge of AP and frequency-dependent AP widening in the presence of Yoda1 + Dbtm were abolished by reverse mode of Na/Ca exchange inhibition in identified Ah-type BRNs. Figure S11: The current density of total K^+^ currents was measured at 400 ms of recording before and after Yoda1 10 nM or Dbtm 100 nM. Averaged data were expressed as mean ± SD, *n* = 15 complete recordings. Figure S12: Effects of KCa1.1 activation by NS11021 on APD and membrane currents observed in the presence of Yoda1 and Yoda1 + Dbtm, respectively, recorded from identified Ah-BRNs. Figure S13: The representative voltage-clamp protocol for Na/K-pump current recording used for Yoda1 10 nM and dobutamine 100 nM alone, or Yoda1 + dobutamine.
