# Supplementary material for: Caution for Multidrug Therapy: Significant Baroreflex Afferent Neuroexcitation Coordinated by Multi-Channels/Pumps Under the Threshold Concentration of Yoda1 and Dobutamine Combination
Source: Biomolecules. 2024 Oct 16;14(10):1311. doi: 10.3390/biom14101311 (PMC11506362; doi:10.3390/biom14101311)

Online only supplemental data

Caution for multidrug therapy: Significant baroreflex afferent neuroexcitation coordinated by multi-channels/pumps under the threshold concentration of Yoda1 and dobutamine combination

Yin-zhi Xu[1,2]*, Zhao-yuan Xu[1,2]*, Hui-xiao Fu[1], Mao Yue[1], Jia-qun Li[1], Chang-peng Cui[1], Di Wu[1,3], Bai-yan Li[1,2]✉

Supplemental Figures:

Figure S1 Gap-free protocol for inward current recording under the voltage-clamp mode with -60 mV holding.

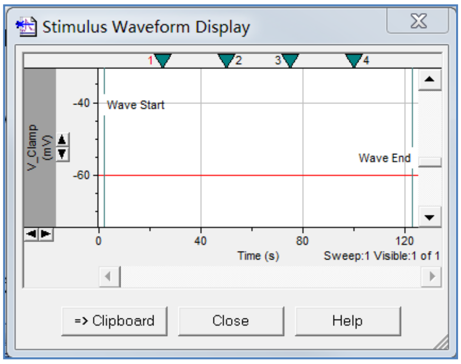

Figure S2 Electroneurography (ENG) of aortic depressor nerve (ADN) at 1.0V stimulation (control) and in the presence of Yoda1 10 nM (Yoda1/10) or Dobutamine 100 nM (Dbtm/100). The distance and time spend between stimulus and recording electrodes placed on the aortic depressor nerve (ADN) was 18 mm of this particular preparation and ~0.47 ms, respectively, so the calculated conduction velocity (CV) was ~38.29 m/s at body temperature (~34°C), that is significantly faster than that was measured at room temperature (18-22 m/s, ~22°C). Arrowhead points out the stimulus artifacts.

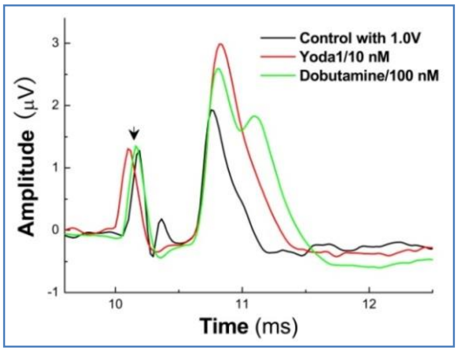

Figure S3 Compound action potential recorded by using electroneurography (ENG) of aortic depressor nerve (ADN) at 6.0V stimulation (control). This is a representative recording of C-volley (C-type compound action potential) evoked at 6V stimulation and majority of C-volley was observed at 5V stimulation and beyond with conduction velocity (CV) less than 2.0 m/s at body temperature that is significantly slower (less than 1.0 m/s in all cases) than that is observed in the room temperature.

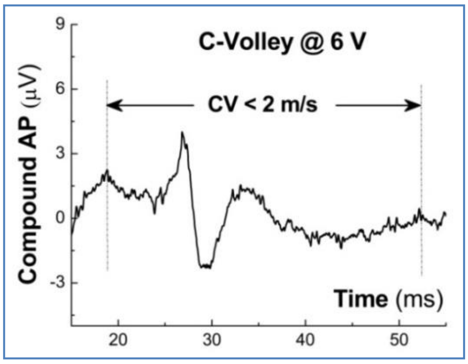

Figure S4 Action potential (AP, black) and membrane current plotted as the function of membrane voltage/derivatives (dV/dt, blue) recorded in identified myelinated Ah-type baroreceptor neurons (BRNs) isolated from adult female rats. The characteristic parameters for this identification include low AP firing threshold, fast depolarization (upward portion of derivative) and repolarization (downward portion of derivative) with significant repolarization hump (vertical red dot line) were well predicted as brief AP duration compared with A- and C-types. the first and second arrowheads mean membrane charging beginning and end with very gentile bridge balance under current-clamp configuration, suggesting the high quality of patch.

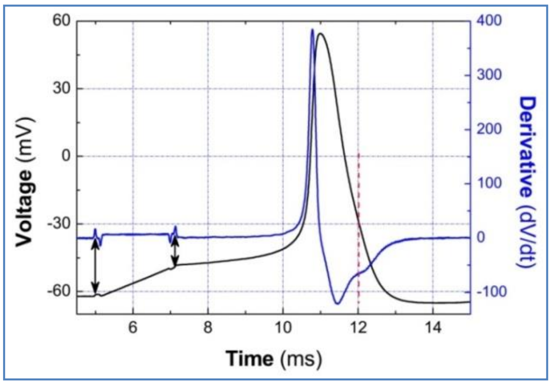

Figure S5 The illustration of the voltage-clamp protocol for step current depolarization to elicit the repetitive discharge of action potential. The stimulus intensity (pA) was selected to elicit only single action potential by first step and the interval between steps was determined upon the excitability (repetitive discharge capability) of each tested cell for about 10, 15, and 20 pA.

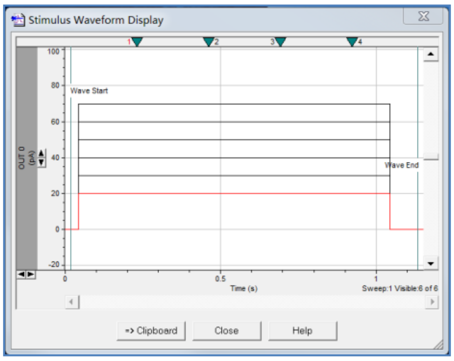

Figure S6 The representative recording of repetitive discharge of action potential in the presence of Yoda1 or Dobutamine. Under the same depolarization currents, the repetitive discharge was recorded in the presence of Yoda1 10 nM (upper panel) or Dobutamine 100 nM (lower panel) in identified Ah-type baroreceptor neurons isolated from adult female rats. The scale bars were applied for all recordings.

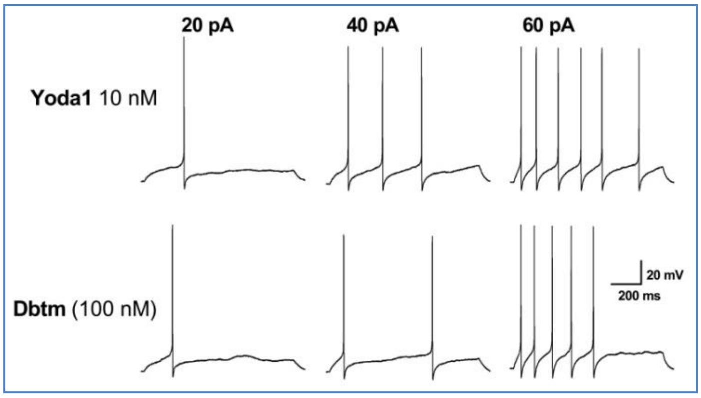

Figure S7 The representative voltage-clamp protocols for ramp recordings used for Yoda1 10 nM and Dobutamine 100 nM alone (left) or Yoda1 10 nM + Dobutamine 100 nM (right).

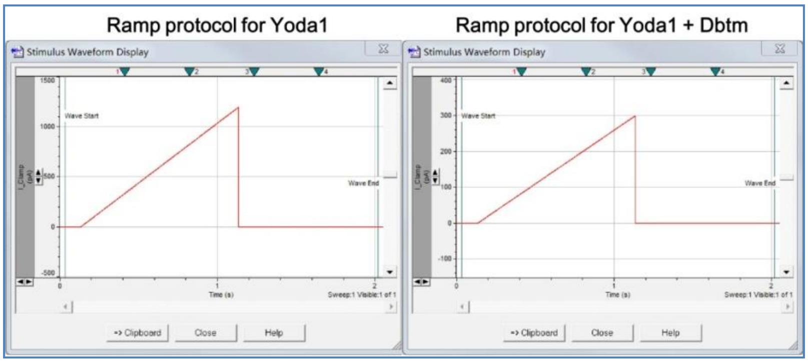

Figure S8 Effects of Yoda1 along (black) and combination of Yoda1/Dbtm (red) on reparative discharge and the Sag potential/hyperpolarization recorded in the Ah-type baroreceptor neurons isolated from adult female rats. The sag potential was recorded using the protocol illustrated as left

panel and representative recording shown as right panel (Trace 1 & 4). Averaged data were presented as mean ± SD, n = 13 complete recordings, $*P < 0.05$ and $**P < 0.01$ *vs*. Yoda1 along.

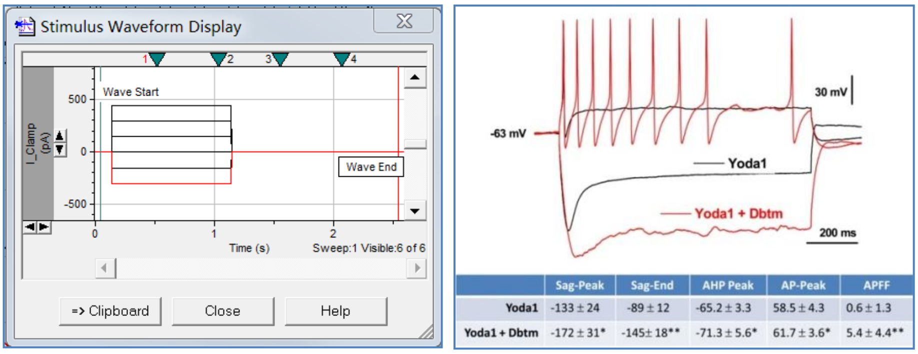

	Sag-Peak	Sag-End	AHP Peak	AP-Peak	APFF
Yoda1	-133 ± 24	-89 ± 12	-65.2 ± 3.3	58.5 ± 4.3	0.6 ± 1.3
Yoda1 + Dbtm	-172 ± 31*	-145± 18**	-71.3 ± 5.6*	61.7 ± 3.6*	5.4 ± 4.4**

Figure S9 The superimposition of the last recording traces elicited by step protocol showing in the figure 3 in the presence of Yoda1 as control and the Yoda1/Dbtm combination.

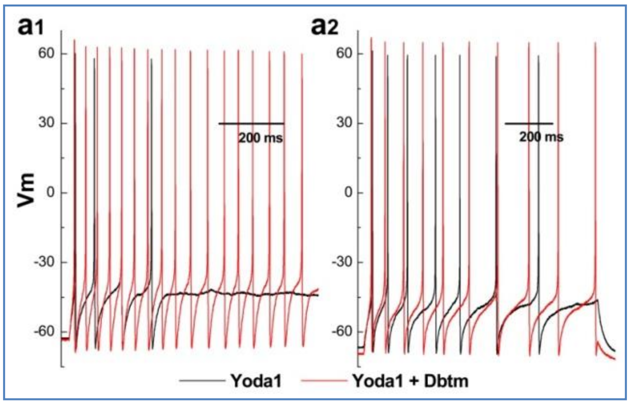

Figure S10 Repetitive discharge of AP and frequency-dependent AP widening in the presence of Yoda1 + Dbtm were abolished by reverse mode of Na/Ca exchange inhibition in identified Ah-type BRNs. Repetitive discharge of AP was elicited by step depolarization in the presence of Yoda1 + Dbtm and this combination/with Kb-r7943, a selective antagonist for Na/Ca exchanger (NCX), and complete recordings in one cell was included for further analysis. (A) Representative recording in the presence of Yoda1 + Dbtm; (B) APs pointed by the colored arrowheads were superimposed; (C) Representative recording in the presence of Yoda1 + Dbtm after pretreatment the tested neurons with 1.0 μM Kb-r7943; (D) Superimposition of APs as pointed by the colored arrowheads. The horizontal bars shown in (c) and (d) also applied for (a) and (b).

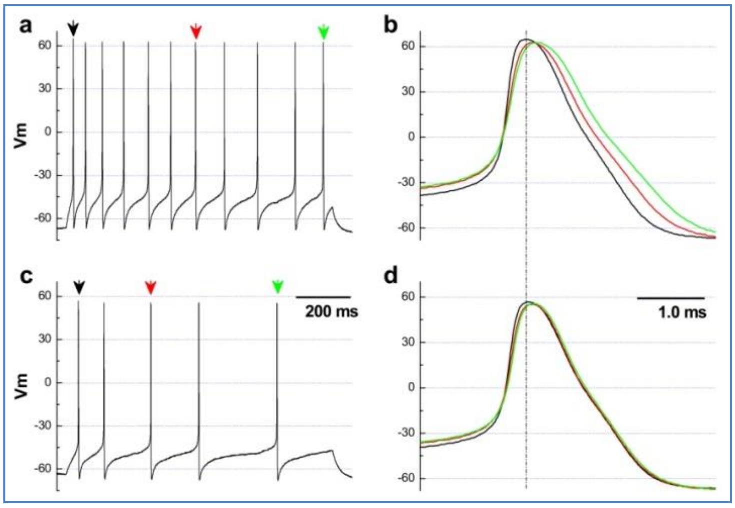

Figure S11 The current density of total K^+ currents were measured at 400 ms of recording before and after Yoda1 10 nM or Dbtm 100 nM. Averaged data were expressed as mean ± SD, n = 15 complete recordings.

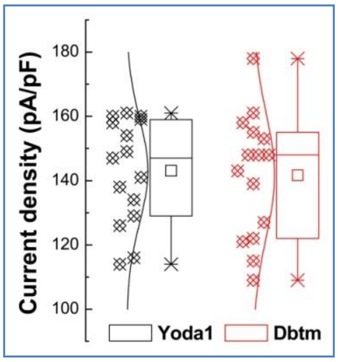

Figure S12 Effects of KCa1.1 activation by NS11021 on APD and membrane currents observed in the presence of Yoda1 and Yoda1 + Dbtm, respectively, recorded from identified Ah-BRNs. (A) Superimposition of the last AP observed in the presence of Yoda1, Yoda1/Dbtm combination, and the combination with NS11021 100 nM; (B) Membrane currents plotted as the function of membrane voltage created form those APs in (a); (C and D) Summary for outward peak of membrane current/downward portion and APD_{50}. Averaged data were expressed as mean ± SD, n = 5 complete recordings from 4 preparations. $^{**}P < 0.01$ *vs*. combination. $^{\#\#}P < 0.01$ *vs*. Yoda1. The scale bar in (a) is also applied for (b).

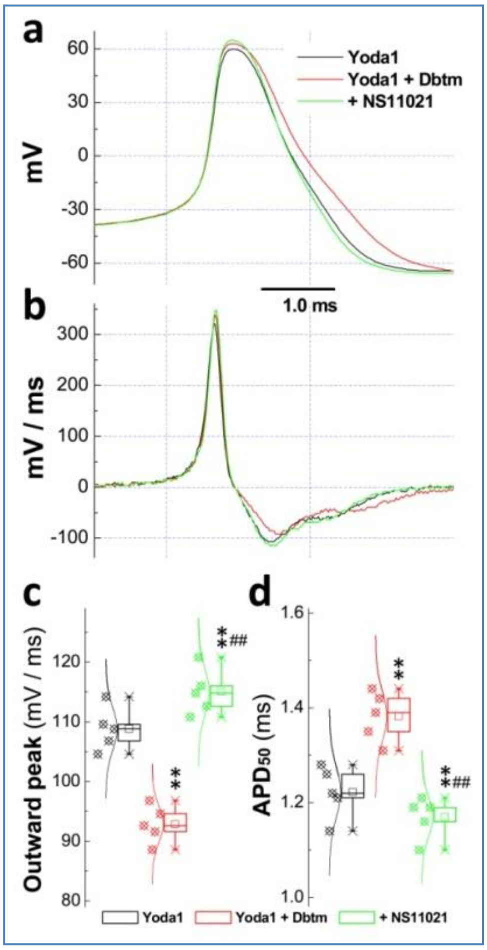

Figure S13 The representative voltage-clamp protocol for Na/K-pump current recording used for Yoda1 10 nM and dobutamine 100 nM alone, or Yoda1 + dobutamine.

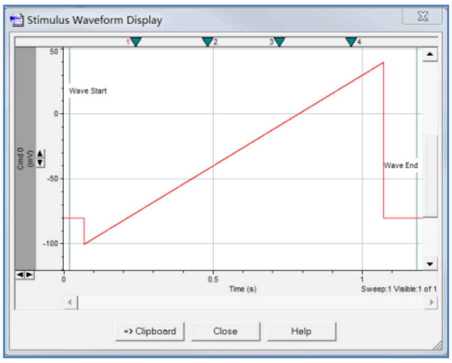

Supplement: Supplementary file 1 [file biomolecules-14-01311-s001.zip › biomolecules-3104765-supplementary.pdf]
